# Systems biology-driven identification of biomarkers and significant pathways in radiation-induced hormone-sensitive cancers

**DOI:** 10.1007/s12672-025-03892-3

**Published:** 2025-11-04

**Authors:** Suvitha Anbarasu, Sathyanarayan Balaji, Sudha Ramaiah, Anand Anbarasu

**Affiliations:** 1 Department of Biotechnology, School of Bioscience and Technology (SBST), Vellore Institute of Technology (VIT), Vellore, Tamil Nadu 632014 India; 2 Medical and Biological Computing Laboratory, School of Bioscience and Technology (SBST), Vellore Institute of Technology (VIT), Vellore, Tamil Nadu 632014 India; 3 Department of Biosciences, School of Bioscience and Technology (SBST), Vellore Institute of Technology (VIT), Vellore, Tamil Nadu 632014 India

**Keywords:** Ionizing radiation, Nucleic acid damage, Hypoxia, Differential expression, Survival

## Abstract

Radiation-induced damage to nucleic acid and other important cellular constituents could likely cause cancer. The present study has analyzed mutated genes with dysregulated expression signatures due to radiation. We have predicted biomarkers specific to hormone-sensitive cancers (HSCs) namely breast cancer (BC), prostate cancer (PC), ovarian cancer (OC), and endometrial cancer (EC). The study has also attempted to prospect the pathways associated with HSCs and radiation exposure through network analysis and functional enrichment analysis. An overlap of important cell cycle pathways, DNA binding, and transcription activity pathways were found in comparison with HSCs and the radiation hallmark pathways. Hub genes *TNF*,* STAT3*,* CTNNB1*, and *MYC* in BC; *IL1B*,* CTNNB1*,* ESR1*, and *SRC* in PC; *CTNNB1*,* BRCA1*,* JUN*, and *KRAS* in OC; *PIK3CA*,* PTEN*, and *ESR1* in EC were found as hypoxic signatures due to radiation. Further, the expression survival analysis found *MYC* and *STAT3* in BC, *CTNNB1* in PC, *JUN*, and *BRCA1* in OC, and *ESR1* in EC were significantly dysregulated and further mutational profiling validated *MYC* with 18%, *STAT3* with 2.6%, and all other genes with 4% mutation. Thus, these genes are proposed as radiation-sensitive diagnostic biomarkers in HSCs.

**Supplementary Information:**

The online version contains supplementary material available at 10.1007/s12672-025-03892-3.

## Introduction

The health effects of ionizing radiation have been known for years since the discovery of X-rays. The exposure has led to epilation, skin burns, and tissue damage. Carcinogenesis due to radiation majorly depends on the dosage of the radiation. Although high doses are proven to have cancer risk, low doses are not understood clearly yet they are responsible for causing carcinogenic effects [1]. The response to these radiations can be categorized into three aspects namely, radio-sensitivity (noncancerous effects after high doses), radio-susceptibility (cancerous even at low doses), and radio-degeneration (noncancerous, but attributed to other health hazards) [2]. Radiation exposure could occur either in the workplace or the environment or through diagnostic medicines. Uncovering a universal model on how these radiations are affecting human life at different scales from the molecular level to the organism level is quite complicated. Yet radiobiological data available on the biological responses to radiation give us better insights into understanding the impacts of this radiation on the human body. In general, these radiations are mutagenic and harmful. Fundamentally these radiations damage the DNA resulting in mutations and chromosomal aberrations further resulting in several diseased conditions including cancer. A scientific assumption that prolonged exposure to these radiations with a linear increase in dosages can result in cancer has been defined. Interestingly later the nonlinear responses were also found to be known as non-targeted effects which might be due to epigenetic changes, leading to dysregulation of genes and their expression resulting in cancerous conditions [3].

The current research focuses on studying the major genes affected by the radiation. The pathways associated with these genes were also investigated, thereby understanding the mechanisms through which radiation-induced mutated genes could cause cancer and how they contribute to further consequences of cancer. Among different cancers, we focused only on hormone-sensitive cancers (HSCs) namely, breast cancer (BC), prostate cancer (PC), ovarian cancer (OC), and endometrial cancer (EC). All these cancers are common and frequent and the incidence and death rates keep increasing every year [4]. Ionizing radiation exposure had an increased risk of acquiring breast cancer, especially among females who had a family history of cancer and might already have the pre-deposited mutations even smaller doses of radiation can contribute to acquiring breast cancer.

Factors including hormones, lifestyles, environmental factors, and genetic factors can cause breast cancer [5]. The association between occupational exposure to radiation and breast cancer was found to increase the risk of breast cancer among healthcare workers. Additionally, long-duration exposure to non-ionizing radiations can slowly lead to cancer in the future [6].

Similar to breast cancer, ionizing radiation plays a major role in ovarian cancer also. *BRCA1* and *BRCA2* are well-established significant mutations in ovarian cancer. These genes are found to be mutated upon DNA damage by ionizing radiations and are involved in disrupting major cellular functions and pathways [7]. The ovary normally has a high probability of acquiring radiation-induced damage as it has female germline cells. The radiation-induced genetic mutations can be carried over to the next generations making the offspring more susceptible to cancer and vulnerable to radiation-based diagnosis [8]. Radiation exposure can cause oncologic conditions with cervical and endometrial cancers with improved survival potency in these cancers [9]. Radiation therapy for cervical cancer was found attributing to endometrial polyps which can later become endometrial cancers [10]. Prostate cells normally have pro-oncogenic factors which makes them more susceptible to acquiring cancer [11]. Additionally, diagnostic procedures involving radiation have also shown an increased risk of prostate cancer [12].

The cancer cells can alter various signaling pathways to stress response and can easily adapt to the tumor microenvironments (TME). One of the TME conditions is deprived oxygen levels in the cell known as hypoxia which can further help in the angiogenesis process for cancer cells. The condition also contributes to major cancer pathways including the mTOR pathway, PI3K pathway, and AMP pathways. Hypoxia also helps in the regulation of autophagy to resist interferon-gamma and tumor necrosis factor-induced cytotoxicity [13]. Hypoxia and Oxic cells are both important signature conditions associated with radiation exposure. Further, the need to identify a biomarker for cancers induced by radiation is important to decide whether to proceed with radiation therapy as a treatment option. The following study employs a systems biology approach to discover the major pathways associated with the mutated genes as well as to elucidate the key biomarkers in HSCs that can act as a diagnostic marker and in the selection of treatment strategies to treat the associated HSCs.

## Materials and methods

### Data retrieval

The Comparative Toxicogenomics Database (CTD), 2023 (https://ctdbase.org/) is a platform that provides insights into the effect of environmental exposure on the health of human. It is a manually curated database that interrelates data on chemicals, genes, phenotypes, diseases, and exposure content derived from published studies [14]. The genes associated with neoplasm induced by radiation were retrieved by searching the keywords “neoplasm”, “radiation” and “cancer” through the accession ID D009381 available in the disease tab followed by the genes tab on the CTD home page. The Cancer Genetics Web database, 2020 provides comprehensive information about genes and their genetic mutation associated with cancer (http://www.cancerindex.org/geneweb/) [15]. The mutated genes in each of the cancers BC, PC, EC, and OC were manually retrieved by searching concerned cancer under each chromosome available at the chromosome icon on the homepage. The redundant genes from CTD were excluded and the genes which are uniquely present in the mutation gene dataset were selected for further studies. The gene signatures that are altered due to radiation exposure under oxic and hypoxic conditions were retrieved from RadiationGeneSigDB at (https://github.com/vmsatya/RadiationGeneSigDB) [16]. The hallmark genes up-regulated and down-regulated in response to ultraviolet (UV) radiation were obtained from the Gene Set Enrichment Analysis (GSEA) database (https://www.gsea-msigdb.org/gsea/index.jsp) [17]. The radiation datasets acquired from Radiation Gene Sig DB and GSEA databases were used for comparative validations of the mutated gene dataset.

### Hub gene identification

The gene set filtered out from the mutation dataset for all four cancers was submitted to STRING v 12.0, 2023 (Search Tool for the Retrieval of Interacting Genes) with a confidence score of 0.7 and based on evidence of the nodes, gene interaction networks (GIN) were constructed. The STRING database serves as a repository of computationally predicted, knowledge transfers across different organisms and interaction information from primary databases [18]. For EC, there were less significant genes in STRING, thus two-fold options were used to obtain more genes associated with the input genes. The gene dataset from the GSEA database was also subjected to GIN construction. Cytoscape a software platform for analyzing the GIN was used For the identification of hub genes [19]. The GIN from STRING was visualized and analyzed for hub genes using the cytoHubba plugin in Cytoscape which helps in the exploration of fragile and important nodes in a network [20]. The hub genes were identified using topology and centrality algorithms such as degree, Edge Percolated Component (EPC), closeness, and betweenness, from the GIN of BC, PC, EC, OC, and GSEA gene sets respectively.

### Network clustering

The network clustering for the GIN of the cancers and GSEA gene dataset was performed in Cytoscape using the MCODE (Molecular Complex Detection) cluster algorithm that detects densely connected regions in large GIN that may represent molecular complexes [21]. The optimum clusters were obtained with k-means value >3, clustering score ≥ 5.0, and number of nodes >10 parameters in MCODE.

### Functional enrichment analysis

The functional enrichment for all the clusters obtained from MCODE was profiled for Gene Ontology (GO) and pathway enrichment using g: Profiler, 2023 webserver (https://biit.cs.ut.ee/gprofiler/gost). It is a high-throughput web server designed for the characterization of manipulating genes, including their ontology, pathway, and transcription factor binding site enrichment [22]. For the current study, the KEGG pathway enrichment analysis was performed, where the overlapping of pathways associated with the genes in the HSCs and the pathways associated with mutated genes driven by radiation demonstrates that the genes may follow similar mechanisms to induce cancer. The GO enrichment of the clusters involves the annotation of biological process (BP), molecular function (MF), and cellular component (CC). Similarly, the overlapping gene ontologies were observed confirming the mechanisms through which the genes contribute to the HSCs. Furthermore, the pathway enrichment was performed with Kyoto Encyclopedia of Genes and Genomes (KEGG) database sources in the web server [23]. The analysis results from this server were validated using the hypergeometric P-values to identify the significant terms corresponding to the cluster genes [24].

### Comparative analysis

The oxic and hypoxic gene signatures in the radiation gene dataset obtained from the RadiationGeneSigDB were used to compare and see if the cluster genes of different cancers are oxic/hypoxic gene signatures. This was performed by constructing a Venn diagram for oxic genes, and hypoxic genes in correlation to the cluster genes using Bioinformatics and evolutionary genomics webserver (BEG), (https://bioinformatics.psb.ugent.be/webtools/Venn/) [25]. Further, the presence of hub genes was also validated for the radiation gene set to find the oxic and hypoxic gene signatures.

### Gene expression analysis

The expression level of hub genes across different tumor tissues taken in this study were analyzed using the TNM plot webserver, 2021 (https://tnmplot.com/analysis/) to assess their regulatory pattern. This is an integrated database built using transcriptome-level datasets to compare gene expression across normal, tumor, and metastatic tissues [26]. This analysis included examining of differential expression of the hub genes obtained for the four cancer types in normal and tumor tissues based on the median expression value of the genes in tumor and normal tissue, median fold change value obtained for each gene in their respective tumor tissue [27].

### Survival prognosis

Survival analysis is a crucial study in medical research, focusing on data derived from clinical outcomes and treatment efficacy. A web portal, cBio Cancer Genomics portal (http://cbioportal.org/), v6.3.5 is an interactive resource to explore the multidimensional cancer genomic data sets used to construct the Kaplan-Meier survival (KM-plot) plot for all the hub genes across the four cancers studied [28]. Due to data insufficiency for EC and OC for survival analysis, the uterus and ovarian cancer dataset studies were combined for the survival curve construction for the hub genes of these corresponding cancers. The KM-plot generates survival curves using Cox proportional hazards analysis to predict survival outcomes [29]. The hub genes were screened based on their default hazard ratio (HR >1.0) and median survival months (MSM) when compared to unaltered gene survival months.

### Validation

The validation for the genes that were screened from the survival analysis was performed by analyzing their mutation profile for each of the genes in their appropriate cancer dataset in the cBioPortal [28]. This analysis was performed to validate the established gene mutations present in the hub genes correlated to radiation exposure according to this study. The OncoPrint feature in the portal displayed the types and extent of genetic alteration in those genes corresponding to their cancer type. The KEGG pathway enrichment analysis was performed for each identified biomarker, allowing for comparative analysis to identify significant pathways related to cancer pathways in the respective HSCs.

## Results

### Retrieved data

The CTD database had 6965 genes under the neoplasms induced by radiation. From the cancer genetics web, 1232 mutated genes were obtained for BC, 622 for PC, 343 for OC, and 41 for EC. The common genes in both databases were taken for network construction which had 700 genes in BC, 343 for PC, 177 for OC, and 24 genes in EC. The GSEA database which had two hallmark gene-sets for upregulated and downregulated genes under UV radiation was also retrieved with 302 genes. The hypoxic and Oxic gene signatures due to radiation contained 3779 and 1144 genes respectively, were retrieved from the RadiationGeneSigDB which were all used for comparative analysis.

### Hub gene identification

The filtered genes were subjected to network construction of high confidence score (0.7) and found a BC network with 658 nodes and 13,886 edges, a PC network with 307 nodes and 3388 edges, an OC network with 142 nodes and 914 edges, and an EC network with 31 nodes and 248 edges (after a two-fold increase in the network). The GSEA UV gene-set network had 204 nodes and 950 edges at the same confidence. The networks were exported to Cytoscape and cytoHubba found the hub genes with four parameters namely degree, closeness, betweenness, and EPC. The Hub gene rank and score with each parameter are documented in Table 1.

**Table 1:** Identified hub gene parameters and scores for four cancers and UV GSEA gene dataset

Data type	Hub genes	Function	Topology scores		Centrality scores	
			Degree	EPC	Betweenness	Closeness
BC	*TP53*	Multifunctional transcription factor (TF)	414.00	188.28	66052.91	419.25
	*AKT1*	AKT – kinase – Cellular regulation	304.00	186.21	26504.35	391.92
	*IL6*	IL6 signalling pathway – Cytokine regulation	286.00	183.87	15282.55	379.08
	*TNF*	Tumor cell growth regulation	274.00	182.41	16568.99	375.25
	*STAT3*	Signal transducer and acivation	274.00	184.04	16178.53	381.00
	*CTNNB1*	Wnt signalling pathway – downstream regulator	270.00	182.57	35273.93	381.33
	*MYC*	Non- specific DNA binding TF	256.00	182.70	17974.41	376.25
PC	*TP53*	Multifunctional transcription factor (TF)	194.00	45.40	20937.13	191.92
	*IL6*	IL6 signalling pathway – Cytokine regulation	154.00	44.38	8338.02	179.83
	*IL1B*	Pro -inflammatory cytokine regulation	124.00	41.74	6492.41	170.83
	*CTNNB1*	Wnt signalling pathway – downstream regulator	118.00	39.98	9869.27	171.75
	*ESR1*	Multifunctional nuclear hormone receptor	98.00	39.27	6872.02	166.42
	*SRC*	Non-protein receptor tyrosine kinase regulation	98.00	38.97	5363.68	164.67
OC	*TP53*	Multifunctional transcription factor (TF)	100.00	31.26	5890.70	88.20
	*CTNNB1*	Wnt signalling pathway – downstream regulator	60.00	27.59	3299.99	77.03
	*BRCA1*	Ubiquitin-protein ligase – DNA damage responses	60.00	28.61	1541.77	75.20
	*JUN*	Multifunctional TF – binding to DNA	52.00	27.35	1864.43	74.95
	*HSP90AA1*	Multifunctional molecular chaperone	44.00	24.28	1625.34	70.82
	*KRAS*	Ras protein driven oncogenic event regulation	44.00	25.09	1634.32	72.20
	*ERBB2*	Protein tyrosine kinase – transcriptional regulation	42.00	26.05	1592.43	72.70
EC	*PIK3CA*	Phosphoinositide-3-kinase (PI3K) phosphorylates phosphatidylinositol (PI)	38.00	82.94	82.94	24.33
	*PTEN*	Dual-specificity protein phosphatase	38.00	93.29	93.29	24.33
	*PIK3R1*	Phosphoinositide-3-Kinase Regulatory Subunit 1	38.00	123.54	123.54	24.33
	*ESR1*	Multifunctional nuclear hormone receptor	36.00	128.90	128.90	24.00
	*TP53*	Multifunctional transcription factor (TF)	34.00	103.11	103.11	23.50
	*PIK3R2*	Phosphoinositide-3-Kinase Regulatory Subunit 2	30.00	46.85	46.85	22.17
	*ESR2*	Multifunctional nuclear hormone receptor	28.00	66.71	66.71	21.83
	*ARID1A*	Involved in transcriptional activation and repression	20.00	43.02	43.02	19.67
UV GSEA	*PTEN*	Dual-specificity protein phosphatase	54.00	26.54	3752.34	82.92
	*IL6*	IL6 signalling pathway – Cytokine regulation	50.00	26.24	2371.20	80.92
	*FOS*	Nuclear phosphoprotein – regulating cell signalling	44.00	25.31	2753.09	80.73
	*CASP3*	Thiol protease - major effector caspase in apoptosis	42.00	25.14	3036.14	78.22
	*CAV1*	Scaffolding protein within caveolar membranes	42.00	23.61	3490.21	78.58
	*NFKB1*	NF-kappa-B, multifunctional TF – cell regulation	32.00	23.54	1878.57	77.10
	*MYC*	Non- specific DNA binding TF	42.00	24.66	-	77.73
	*ERBB2*	Protein tyrosine kinase – transcriptional regulation	42.00	25.82	-	77.82

### Clustered subnetworks

**Fig. 1:**
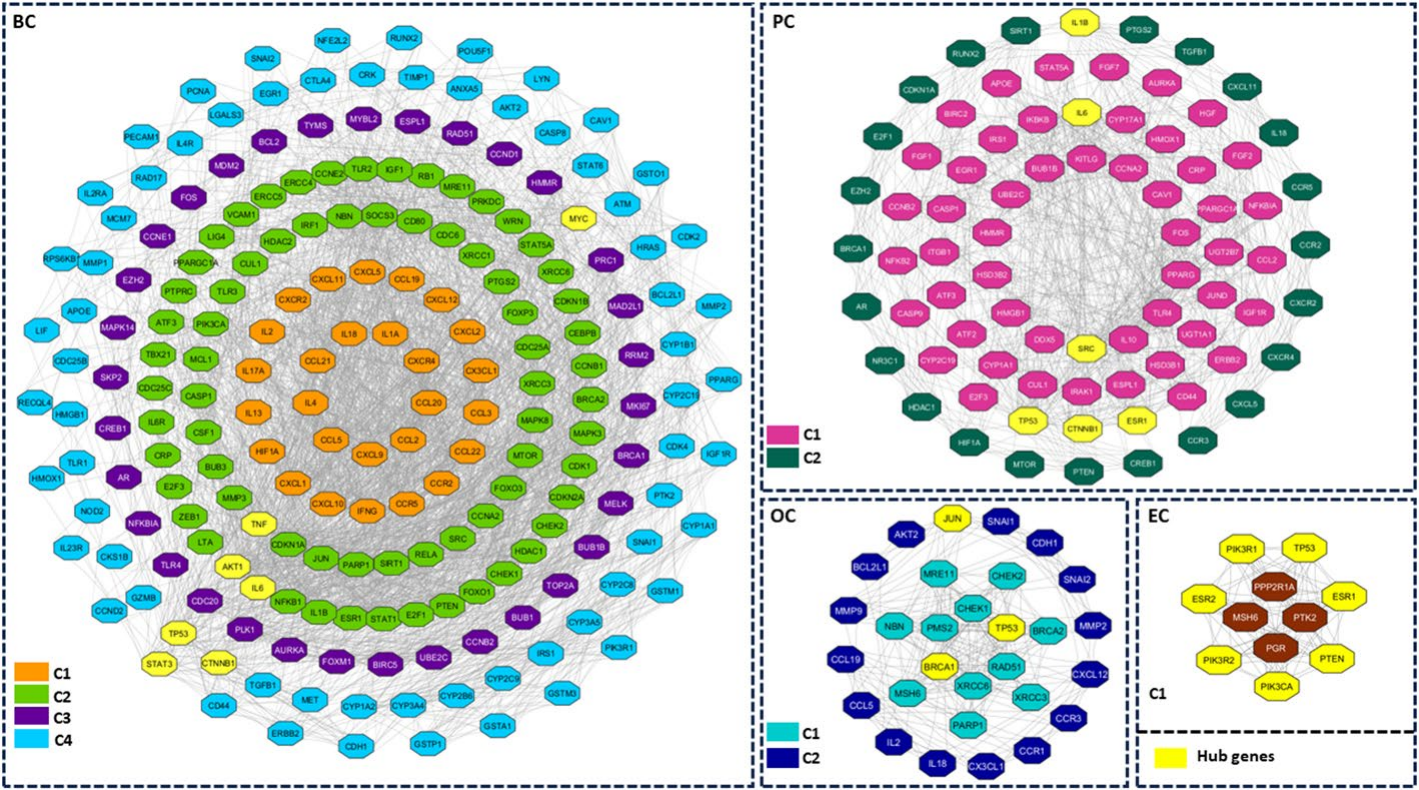
Clusters in HSC. BC - Breast Cancer; PC - Prostate Cancer; OC - Ovarian Cancer; EC - Endometrial Cancer; Hub genes are represented in yellow color

The BC network found four clusters, cluster 1 (C1) with 27 nodes and 542 edges and a clustering score of 20.846, cluster 2 (C2) with 72 nodes and 1128 edges and a score of 15.887, cluster 3 (C3) with 34 genes and 478 edges and a score of 14.485, and cluster 4 (C4) with 69 nodes and 622 edges and a score of 9.147. The PC network found two clusters, C1 with 55 nodes and 498 edges and a score of 9.222, and cluster C2 with 24 nodes and 184 edges and a score of 8.0. The OC network found two clusters, C1 with 13 nodes, and 142 edges and a score of 11.833, and C2 with 16 nodes and 184 edges and a score of 8.0. The EC network found one cluster with 11 nodes and 86 edges and a score of 8.6. All the subnetworks are represented in Fig. 1.

### Functional enrichment

**Fig. 2:**
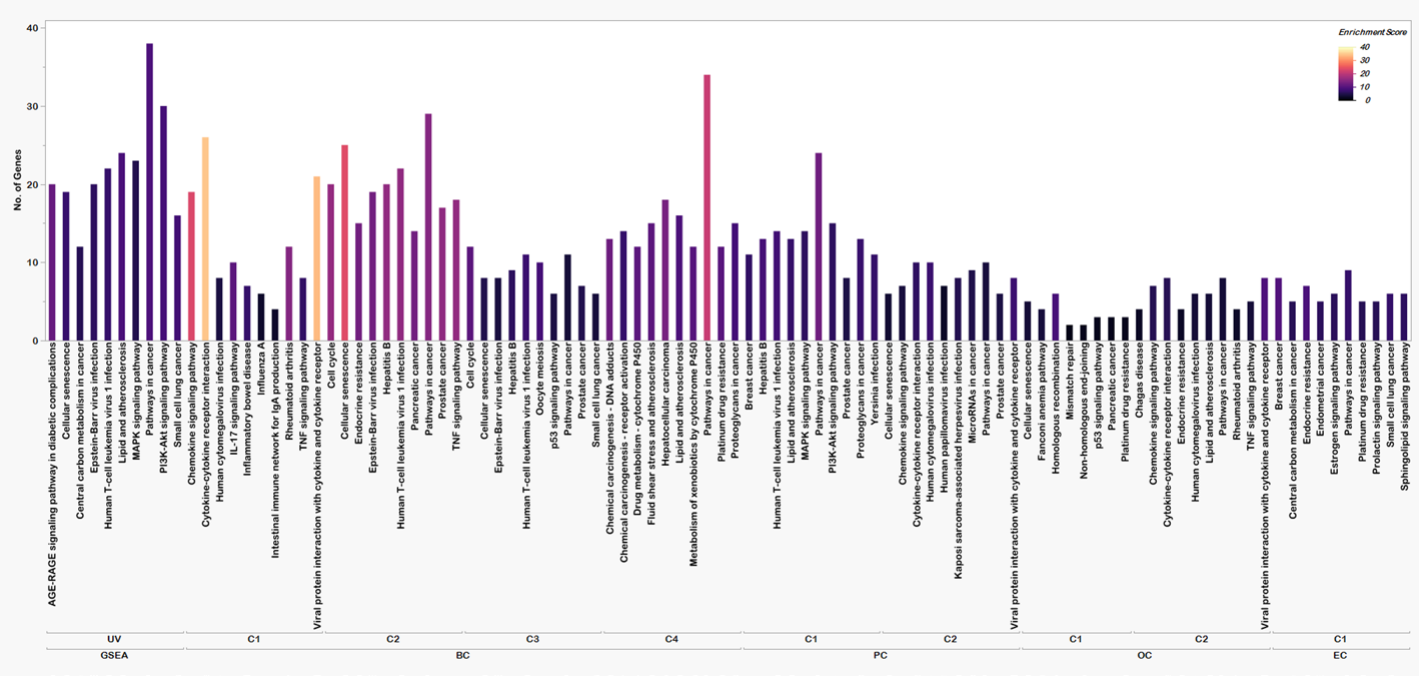
KEGG pathway analysis for HSCs. The X-axis shows the enriched pathways in different clusters of all four cancers and for UV radiation-based pathways, the Y-axis shows the number of genes involved in each of the pathways and the color range from dark purple to red to peach represents the enrichment score of each pathway (Low to high)

**Fig. 3:**
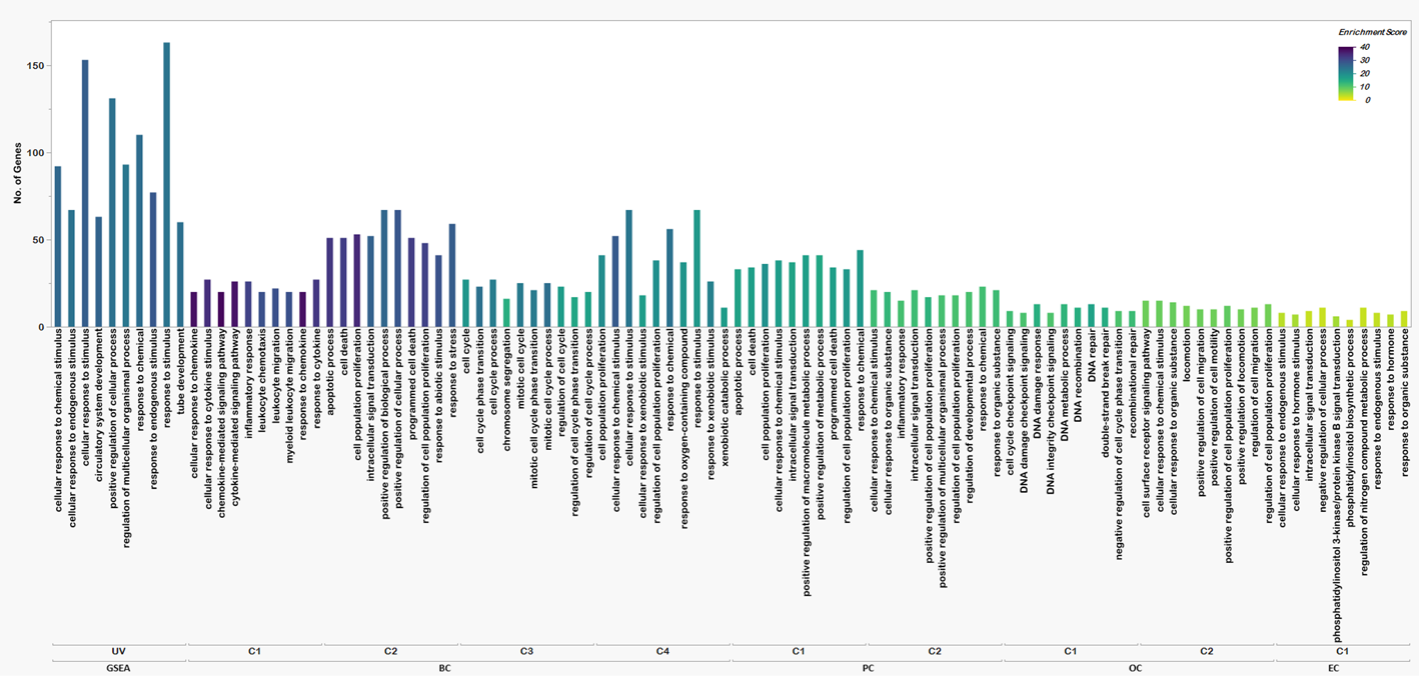
Bioprocess in HSC clusters and UV hallmark genes. The X-axis shows the enriched bioprocesses in different clusters of all four cancers and for UV radiation-based bioprocesses, the Y-axis shows the number of genes involved in each of the bioprocesses and the color range from Yellow to teal green to dark violet represents the enrichment score of each pathway (Low to high)

**Fig. 4:**
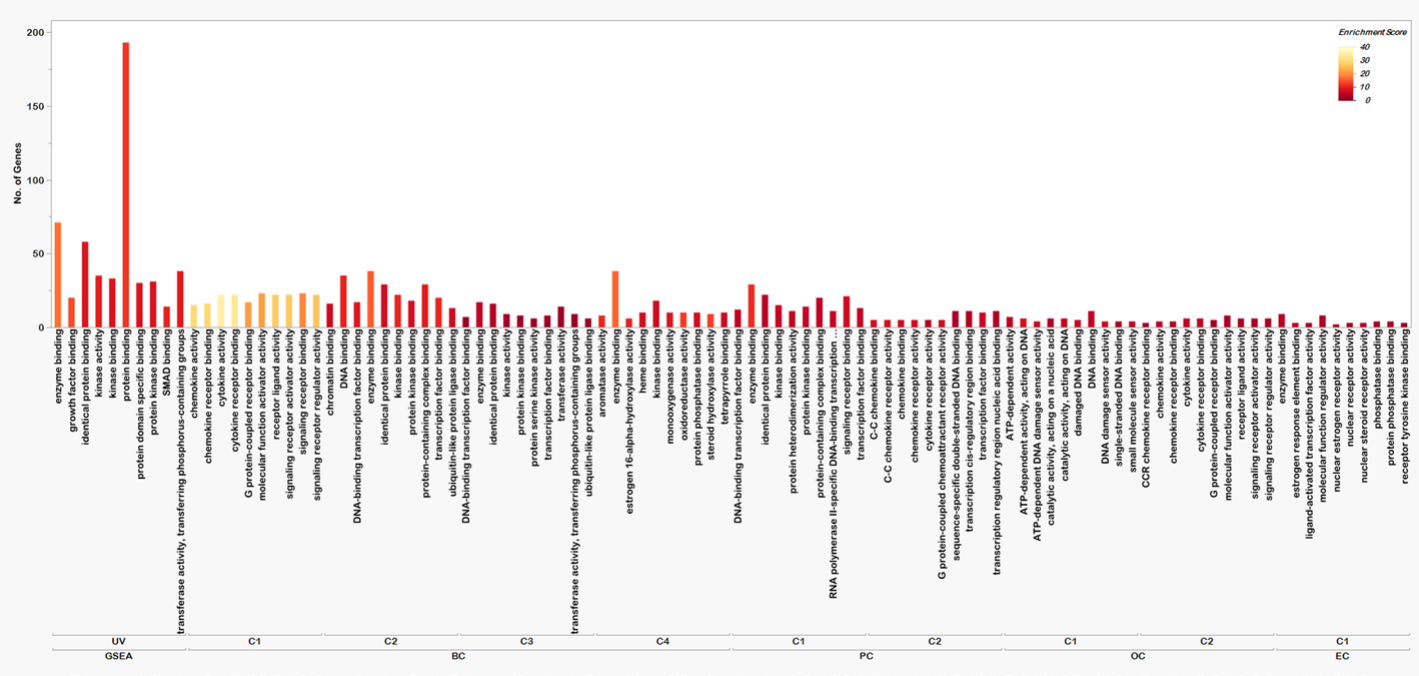
Molecular functions in HSC clusters and UV hallmark genes. The X-axis shows the enriched molecular functions in different clusters of all four cancers and for UV radiation-based molecular functions, the Y-axis shows the number of genes involved in each of the molecular functions, and the color range from maroon to faded yellow represents the enrichment score of each function (Low to high)

**Fig. 5:**
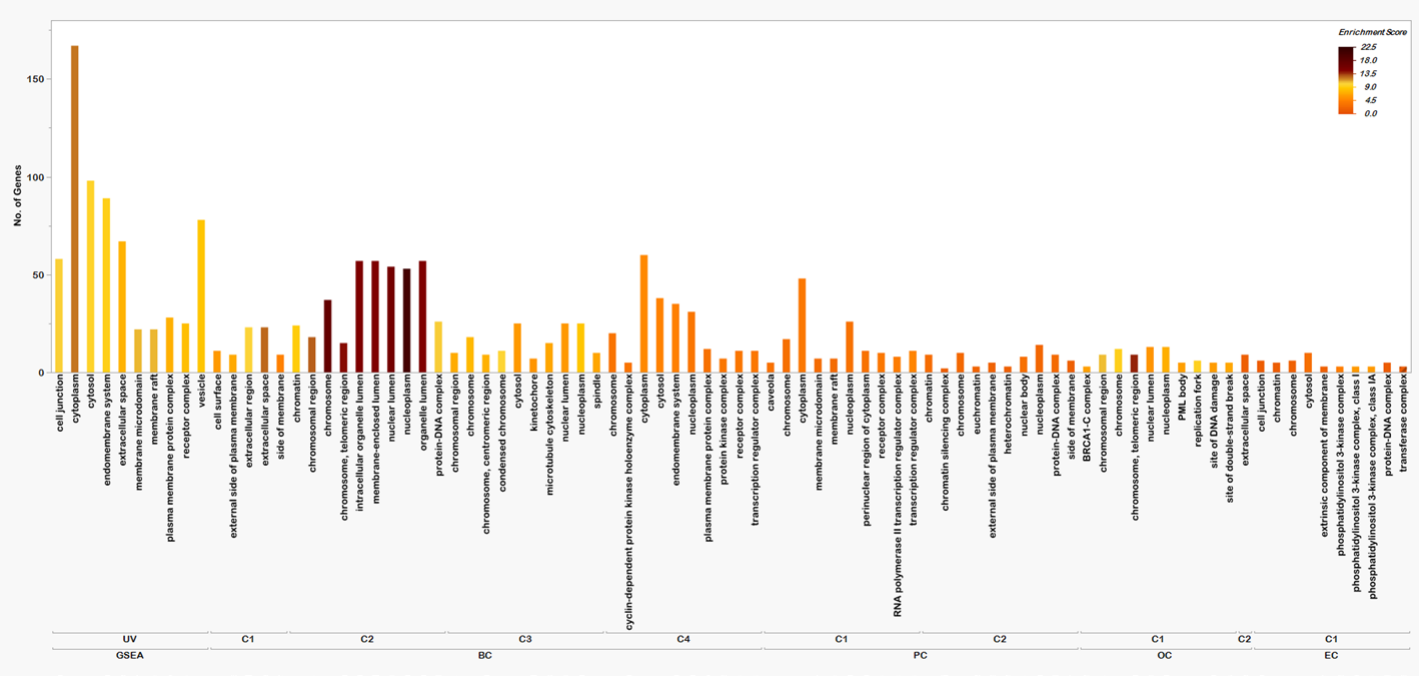
Cellular Components in HSC clusters and UV hallmark genes. The X-axis shows the enriched cellular components in different clusters of all four cancers and for UV radiation-based cellular components, the Y-axis shows the number of genes involved in each of the cellular components and the color range from Orange to yellow to brown represents the enrichment score of each cellular component (Low to high)

The functionally enriched pathways of the four cancers were compared to the functionally enriched pathways of the GSEA gene set and found certain similar pathways. As well as the gene ontologies (GO) were also compared and most of the pathways were related to DNA or protein binding and activity, at most of the membrane regions and in major processes like reaction to stimuli and important regulatory pathways. The BP, MF, CC, and KEGG pathways with the number of genes and the enrichment score (negative logarithm of adjusted P value) are represented in Figs. 2, 3 and 4, and 5 respectively.

### Comparative analysis

The comparison of the presence of the oxic and hypoxic genes in each of the clusters was plotted as a Venn diagram (Figure S1). Based on the comparison between the hub genes, hypoxic and oxic signatures, *TNF*,* STAT3*,* CTNNB1*, and *MYC* were screened for BC, *IL1B*,* CTNNB1*,* ESR1*, and *SRC* were screened for PC, *CTNNB1*,* BRCA1*,* JUN*, and *KRAS* was screened for OC and *PIK3CA*,* PTEN*, and *ESR1* was screened for EC for further expression and survival analysis.

### Gene expression analysis

The hub gene expression analysis of the respective cancers was performed with a TNM plot webserver. This analysis compared the gene expression levels between normal and tumor tissues using the median expression and median fold change values (Table 2). Genes with a median expression level higher than that of normal tissue, combined with a median fold change greater than 1.0, are considered upregulated in the respective tissue. All the hub genes showed different expression profiles across all tumor tissues. In BC, the genes *STAT3*,* CTNNB1*, and *MYC* were upregulated, while TNF was downregulated in tumor tissues. In PC, the genes *ESR1* and *SRC* exhibited overexpression in tumor tissues, whereas *IL1B* and *CTNNB1* showed reduced expression. In EC, *ESR1* was the only hub gene upregulated in uterine tissues, with *PIK3CA* and *PTEN* being downregulated. In OC, *KRAS* was downregulated, whereas the other hub genes *CTNNB1*,* BRCA1*, and *JUN* were upregulated in ovarian tumor tissues.

**Table 2:** Gene expression profile of the hub genes in four cancer types

Cancer type	Gene name	P value	Median		Fold change	Differential expression
			Normal	Tumor		
BC	*TNF*	5.31E-01	110	96	0.87	Downregulated
	*STAT3*	3.01E-12	3026	3728	1.23	Upregulated
	*CTNNB1*	9.52E-15	1878.5	2722	1.45	Upregulated
	*MYC*	9.23E-01	1241	1364	1.1	Upregulated
PC	*IL1B*	9.23E-03	283.5	227	0.8	Downregulated
	*CTNNB1*	1.93E-03	2911	2375	0.82	Downregulated
	*ESR1*	1.28E-01	465	575	1.24	Upregulated
	*SRC*	6.08E-01	303.5	330	1.09	Upregulated
OC	*CTNNB1*	3.65E-09	1548.5	3282	2.12	Upregulated
	*BRCA1*	1.38E-07	109	203.5	1.87	Upregulated
	*JUN*	6.50E-01	3082	2667	1.07	Upregulated
	*KRAS*	7.12E-01	87	86	0.99	Downregulated
EC	*PIK3CA*	6.76E-04	477	300.5	0.63	Downregulated
	*PTEN*	4.64E-03	328	268	0.82	Downregulated
	*ESR1*	2.52E-01	9217	10777.5	1.17	Upregulated

### Survival prognosis

The survival analysis of each hub gene across all four cancer tissues was performed by constructing a KM-plot, which provides hazard ratios, and the median survival means (MSM) associated with each gene. The genes whose hazard ratio (HR) > 1.0 and MSM were shorter than that of the unaltered group were considered crucial for survival in each of the cancers. Based on the above analogy, the genes *TNF*,* STAT3*, and *MYC* were observed to be critical for survival with unaltered group MSM of 169.6 and *CTNNB1* came out to be insignificant in terms of BC. In PC, the genes *IL1B*,* SRC*, and *ESR1* were insignificant but *CTNNB1* was observed to be pivotal for survival MSM of the altered group (56.09) was less than that of unaltered MSM (107.2). Notably, *CTNNB1* was significant with 1.59 h in PC despite being downregulated based on expression analysis. In EC, *ESR1* was the only gene identified as important for survival with an HR of 1.11 and an MSM of 47.67 whereas unaltered MSM was 55.39 in both OC and EC. In OC, *CTNNB1* and *KRAS* were not significant as they lacked notable HR and MSM values. However, the genes *JUN* and *BRCA1* were found to be pivotal for survival. The descriptive details obtained from survival analysis including the number of samples, HR, and MSM of altered and unaltered groups are presented in Table 3. The detailed survival plot based on the probability of survival and survival months of the hub genes in the cancer tissues is illustrated in Figure S2. From this analysis, the genes *STAT3*,* MYC*,* CTNNB1*,* ESR1*,* JUN*, and *BRCA1* were identified for further validation as potential biomarkers.

**Table 3:** Survival analysis of the hub genes

Cancer	Gene	Number at risk	No of unaltered group	Median CI (95%)		HR	P-value
				Altered groups	Unaltered groups		
BC	*TNF*	12	3778	125.9	169.6	1.633	3.08E-05
	*STAT3*	42		100.87		1.34	
	*CTNNB1*	16		NA		0.507	
	*MYC*	801		135.3		1.322	
PC	*IL1B*	8	2190	NA	107.2	1.698	2.37E-02
	*CTNNB1*	84		56.09		1.589	
	*ESR1*	15		158		1.009	
	*SRC*	12		NA		NA	
EC	*PIK3CA*	140	683	67.2	55.39	0.789	1.53E-05
	*PTEN*	72		125.46		0.481	
	*ESR1*	21		47.67		1.111	
OC	*CTNNB1*	17		58.11		0.684	
	*BRCA1*	21		40.97		1.517	
	*JUN*	8		32.85		1.74	
	*KRAS*	54		36.33		NA	

### Validation

The validation for the predicted biomarkers for different cancers with radiation exposure was performed, and genetic alterations of the identified critical survival genes were profiled. The observed genetic alterations were as follows: *STAT3*-2.6%, *MYC*-18%, *CTNNB1*-4%, *ESR1*-4%, *JUN*-4%, and *BRCA1*-4% (Figure S3). Most of these genetic alterations involved gene amplification and fewer deep deletions. The KEGG pathway comparative analysis identified several critical pathways for each cancer type, from which the top 10 pathways, specifically involving the biomarkers, were selected based on enrichment scores. The analysis revealed that *STAT3*, *MYC*, *CTNNB1*, *BRCA1*, *JUN*, and *ESR1* are involved in key pathways of significance relevant to HSCs (Table 4).

**Table 4:** KEGG pathway enrichment analysis data of biomarkers

KEGG pathway enrichment			
Cluster	Enrich-ment score	Pathways	Genes
BC			
C3	11.11	Cell cycle	*ESPL1*, *BUB1B*, *MDM2*, *CDC20*, *CCNB2*, *PLK1*, *BUB1*, ***MYC***, *MAD2L1*, *SKP2*, *CCND1*, *CCNE1*
	5.39	Cellular senescence	*FOXM1*, *MDM2*, *MAPK14*, *CCNB2*, *MYBL2*, ***MYC***, *CCND1*, *CCNE1*
	4.04	Pathways in cancer	*FOS*,* MDM2*, *BCL2*, *RAD51*, *BIRC5*, *NFKBIA*,* AR*, ***MYC***, *SKP2*, *CCND1*, *CCNE1*
	3.23	Chemical carcinogenesis - receptor activation	*FOS*,* BCL2*, *BIRC5*, *AR*, ***MYC***, *CREB1*, *CCND1*
	2.67	PI3K-Akt signaling pathway	*MDM2*, *BCL2*, *TLR4*, ***MYC***, *CREB1*, *CCND1*, *CCNE1*, *BRCA1*
	2.15	MicroRNAs in cancer	*MDM2*, *BCL2*, ***MYC***, *EZH2*, *CCND1*, *CCNE1*, *BRCA1*
C4	21.80	Pathways in cancer	*CDK2*, *RPS6KB1*, *PPARG*,* IL23R*,* IL2RA*, ***STAT3***, *NFE2L2*, *GSTP1*, *MMP2*, *GSTO1*, *CCND2*, *IGF1R*,* ERBB2*, *HMOX1*, *GSTM3*, *GSTA1*, *GSTM1*, *PIK3R1*, *CDH1*, *CDK4*, *AKT2*, *MET*,* CASP8*, *MMP1*, *CRK*,* HRAS*,* STAT6*, *IL4R*,* BCL2L1*, *CKS1B*,* CTNNB1*, *PTK2*, *TP53*, *TGFB1*
	7.42	Chemical carcinogenesis - receptor activation	*RPS6KB1*, ***STAT3***, *GSTO1*, *GSTM3*, *GSTA1*, *GSTM1*, *PIK3R1*, *CYP1A1*, *CYP1A2*, *AKT2*, *HRAS*,* CYP3A4*, *CYP2B6*, *CYP1B1*
	5.57	JAK-STAT signaling pathway	*LIF*,* IL23R*,* IL2RA*, ***STAT3***, *CCND2*, *PIK3R1*, *AKT2*, *HRAS*,* STAT6*, *IL4R*,* BCL2L1*
	5.27	MicroRNAs in cancer	***STAT3***, *CD44*, *CCND2*, *ERBB2*, *HMOX1*, *PIK3R1*, *MET*,* CRK*,* HRAS*,* CDC25B*,* IRS1*, *ATM*,* CYP1B1*, *TP53*
PC			
C1	13.10	Pathways in cancer	*KITLG*,* CCNA2*, *PPARG*,* FOS*,* IL6*, *HMOX1*, *ITGB1*, *IKBKB*,* CUL1*, *FGF1*, *E2F3*, *NFKB2*, *TP53*, *CASP9*, *FGF7*, *ESR1*, *NFKBIA*,* BIRC2*, *FGF2*, ***CTNNB1***, *ERBB2*, *STAT5A*,* HGF*,* IGF1R*
	7.70	Proteoglycans in cancer	*CAV1*, *SRC*,* TLR4*, *DDX5*, *ITGB1*, *TP53*, *ESR1*, *CD44*, *FGF2*, ***CTNNB1***, *ERBB2*, *HGF*,* IGF1R*
	7.00	Breast cancer	*FOS*,* FGF1*, *E2F3*, *NFKB2*, *TP53*, *FGF7*, *ESR1*, *FGF2*, ***CTNNB1***, *ERBB2*, *IGF1R*
	4.97	Prostate cancer	*IKBKB*,* E2F3*, *TP53*, *CASP9*, *NFKBIA*, ***CTNNB1***, *ERBB2*, *IGF1R*
	1.42	Endometrial cancer	*TP53*, *CASP9*, ***CTNNB1***, *ERBB2*
OC			
C1	9.10	Homologous recombination	***BRCA1***, *RAD51*, *NBN*,* XRCC3*, *MRE11*, *BRCA2*
	2.07	Platinum drug resistance	***BRCA1***, *TP53*, *MSH6*
C2	4.03	TNF signaling pathway	*MMP9*, *AKT2*, *CCL5*, ***JUN***, *CX3CL1*
	3.96	Pathways in cancer	*IL2*, *MMP9*, *AKT2*, *MMP2*, *BCL2L1*, *CDH1*, ***JUN***, *CXCL12*
	2.82	Endocrine resistance	*MMP9*, *AKT2*, *MMP2*, ***JUN***
	2.30	Relaxin signaling pathway	*MMP9*, *AKT2*, *MMP2*, ***JUN***
	2.21	Estrogen signaling pathway	*MMP9*,*AKT2*,*MMP2*, ***JUN***
EC			
C1	10.21	Breast cancer	*PTEN*,* PIK3R2*, ***ESR1***, *TP53*, *ESR2*, *PIK3R1*, *PIK3CA*,* PGR*
	9.48	Endocrine resistance	*PIK3R2*, ***ESR1***, *TP53*, *ESR2*, *PIK3R1*, *PTK2*, *PIK3CA*
	7.43	Pathways in cancer	*MSH6*, *PTEN*,* PIK3R2*, ***ESR1***, *TP53*, *ESR2*, *PIK3R1*, *PTK2*, *PIK3CA*
	6.41	Estrogen signaling pathway	*PIK3R2*, ***ESR1***, *ESR2*, *PIK3R1*, *PIK3CA*,* PGR*
	6.06	Prolactin signaling pathway	*PIK3R2*, ***ESR1***, *ESR2*, *PIK3R1*, *PIK3CA*
	5.34	Proteoglycans in cancer	*PIK3R2*, ***ESR1***, *TP53*, *PIK3R1*, *PTK2*, *PIK3CA*
	5.27	Chemical carcinogenesis - receptor activation	*PIK3R2*, ***ESR1***, *ESR2*, *PIK3R1*, *PIK3CA*,* PGR*
	4.86	Thyroid hormone signaling pathway	*PIK3R2*, ***ESR1***, *TP53*, *PIK3R1*, *PIK3CA*

The genes represented in bold are the identified hub genes involved in the respective pathways

## Discussion

Cancer is a multifactorial disease that can be influenced by ionizing and non-ionizing radiation. The United Nations Scientific Committee on the Effects of Atomic Radiation (UNSCEAR) report suggested that ionizing radiations promote the accelerated growth of pre-cancerous clones, and these changes are usually driven by the oxygen conditions of the cells [30, 31]. Thus, the current study has attempted to understand the functional pathway mechanisms through which these radiations might induce cancer as well as the prediction of a diagnostic biomarker from aspects of oxic and hypoxic conditions of a cell. The biological mechanisms through which these radiations could contribute to cancer are currently under research. Predicting a radiation-induced cancer biomarker in cancer could be of great assistance in understanding the role of radiation in cancer.

**Fig. 6:**
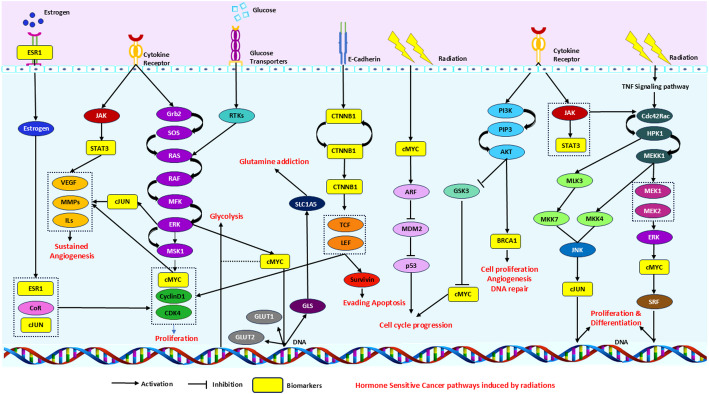
The pathways prominent in radiation-induced HSCs specific to the identified biomarkers. The hub genes are represented in yellow color

The mutated gene list retrieved from the cancer genetics web was encompassed with the radiation-induced neoplasms gene set. The hub genes for each cancer were identified (Table 1) and the functional enrichment analysis found the presence of the hub genes in various important pathways driving cancer progression (Fig. 6).

Studies on neoplasms induced by ionizing radiations found similar pathways namely cell cycle, cell signaling, and DNA repair mechanisms which correlated with our study [32, 33, 35]. Kinases like MAPK, AKT, and PKA phosphorylated CREB upon radiation induced neuroendocrine differentiation in PC [34] which correlated with this study as well. *ARID1A*, one of the hub genes in EC was studied for its mechanisms in DNA damage leading to EC. The study found that radiation-induced DNA damage is difficult to repair and can significantly contribute to endometrial cancer [36]. From the functional enrichment analysis, it is evident that both photo radiation and ionizing radiation can possibly developing HSCs. Further, the hypoxic and oxic conditions of a cell were considered. Important cellular functions including differentiation, proliferation, and growth are all governed by the hypoxic nature of the cancer cells. Hypoxia is one of the well-known phenomena in radiation-induced damage, thus we correlated the presence of hypoxic gene signatures in our clusters [37]. The hub genes which were hypoxic and oxic were considered for further evaluation.

The expression analysis and survival prognosis of the hub gene in different cancer types were performed to obtain the regulatory signatures of the genes in the specific cancers [38]. It also provides a correlation with the radiation signature of the genes by their expression in each of the cancers. Direct ionization or the formation of reactive oxygen species (ROS) can lead to cancer progression [39–41].

Radiation exposure has effects on gene expression in breast cancer cells [42, 44]. While undergoing radiotherapy hypoxic cells tend to resist radio sensitization due to reduced oxygen supply in the TME resulting in disruption of vasculature in the nearby cells, making them hypoxic and resistant to radiation therapy [43]. The identified genes in PC were found to be aiding in the process of hypoxic cell formation, which in turn could lead to radio sensitization of the tumor cells [45]. In EC, it was found that ionizing radiations activate the pro-oncogenic proteins and signaling pathways, protein kinase B (*AKT*), and nuclear factor kappa B which aids tumor cell growth [46]. Thus, the identified hub genes might contribute to tumor. Hypoxic genes were also found associated with the cell cycle, and DNA damage response pathways influencing the radio-sensitivity [47]. An OC study found hypoxic genes were involved in vasculo-genesis, cell growth pathways, and cell metabolism [48] correlated with the identified hub genes in OC.

Investigations on expression of certain genes in the concerned cancer patients unveiled that elevated gene expression correlates with diminished survival rates, as evidenced by survival plots and HR exceeding 1 [49, 50]. In BC, *STAT3* and *MYC* had an HR of 1.63 and 1.32 respectively from which *STAT3* is comparatively more hazardous than *MYC* with a median survival of 100.87 months. In PC, the upregulated genes *ESR1* and *SRC* were not significant enough in survival analysis but the downregulated gene *CTNNB1* was significant with 1.59 h and 56.09 median survival months (Table 3). This indicates that the downregulated genes could also significantly contribute to the survival prognosis of cancer [51, 52]. Whereas in EC, the overexpressed gene *ESR1* came out to be significant in terms of survival with 47.67 months survival rate and 1.11 h. Further analyzing the OC survival curves, *JUN* and *BRCA1* were prominent for their survival parameters with limited survival months of 32.85, 36.33, and 1.74, 1.52 h respectively. Based on this analysis, the genes *STAT3*,* MYC*,* CTNNB1*,* ESR1*,* JUN*, and *BRCA1* emerged as pivotal genes, indicating their significant impact on each cancer type in response to radiation exposure.

Upregulation of *STAT3* in association with different cytokines was essential in regulating various processes in promoting cancer including, tumor progression, proliferation, and metastasis. Ionizing radiations can phosphorylate STAT3, activate the JAK/STAT3 pathway, and influence the downstream targets associated with STAT3 [53, 54]. Similarly, MYC is a multifunctional gene that can bind to various cell proliferation and differentiation-associated genes upon overexpression, inhibit, and promote transformation, stimulate transcription, promote metastasis, alter tumor microenvironment, and contribute to increased drug resistance [55]. Although the role of *CTNNB1* is not well studied in PC, mutations or over-expression of *the CTNNB1* gene were found associated with tumorigenesis. In general, *CTNNB1* is majorly involved in the Wnt/β-catenin pathway whose activation is well established to contribute to cancer [56]. A study on *CTNNB1* in EC cells revealed that the Wnt/β-catenin signaling pathway could be activated when the cells are exposed to radiation and hypoxic conditions where there is nuclear accumulation of β-catenin irrespective of *CTNNB1* mutation in the cells. This study signifies the important role of *CTNNB1* in tumor progression after post-radiation exposure of the cancer cells [57]. In OC, the *JUN* gene, which is part of the *JUN* N-terminal kinase (JNK) signaling pathway, plays a critical role in controlling cell death, survival, growth, and proliferation within the mitogen-activated protein kinase (MAPK) signaling cascade. This gene is frequently activated and is considered essential for OC progression [58]. Another study on the expression of nucleosome assembly protein 1-like 1 (*NAP1L1*) demonstrated that its interaction with hepatoma-derived growth factor (HDGF) in the cytoplasm can stimulate the JUN proto-oncogene. This activation subsequently triggers the proliferation of OC cells through the HDGF/C-JUN signaling pathway. Through this pathway, HDGF enhances JUN expression, promoting cellular mechanisms that lead to OC cell growth and proliferation, highlighting the pathway’s potential role in OC progression [59]. *BRCA1*, a critical tumor suppressor gene and hub in OC, is essential for cellular responses to DNA damage and repair mechanisms that maintain genomic stability. *BRCA1* is directly involved in homologous recombination-mediated repair of double-stranded DNA breaks and coordinates with proteins such as receptor-associated protein 80 and C-terminal binding proteins to form DNA repair complexes, thereby regulating transcriptional repression. Alterations or overexpression of *BRCA1* in OC cells can significantly drive tumor progression, especially under genotoxic stressors like radiation [60–62]. *ESR1* encodes the nuclear transcription factor estrogen receptor α (ERα), which is crucial for the carcinogenic progression of HSC cells. *ESR1* is involved in the E2/ERα signaling pathway, where estrogen (E2) binds to ERα in the nucleus to form the E2-ERα complex. This complex promotes the assembly of the transcription initiation machinery, thereby activating the transcription of target genes. This classical E2/ERα signaling pathway is fundamental to the initiation and progression of EC, driving the expression of genes critical for cell growth and survival [63]. ERα is also integral to multiple critical pathways in cancer, including the JAK/STAT, MAPK, PI3K/AKT/mTOR, and Wnt/β-catenin pathways. Within these pathways, ERα interacts with key regulatory proteins, such as *Bcl-xl*, *PTEN*, *MEK*, and β-catenin, forming intermediate complexes that contribute to both upstream and downstream signaling regulation. These interactions enhance the modulation of cell survival, proliferation, and apoptosis, amplifying the oncogenic processes in EC by coordinating cellular responses across multiple signaling networks [64]. These clinical and experimental studies indicate that the identified hub genes play critical roles in HSC progression, where exposure to photo radiation can markedly alter gene expression in cancer cells, further promoting oncogenesis [65]. Validating the result mutational analysis was performed. Dysregulation of cellular processes facilitated by amplified genes contributes to the progression of cancer [66].

In comparing KEGG pathway enrichment results across four cancer types, several key pathways consistently emerged. Specifically, enrichment data showed the involvement of *MYC* and *STAT3* in BC, *CTNNB1* in PC, *BRCA1* and *JUN* in OC, and *ESR1* in EC. Notably, experimentally validated pathways included the PI3K-AKT signaling pathway, where *MYC* plays a central role, and the JAK-STAT signaling pathway involved *STAT3*. An in-vitro study on prostate cancer cells exposed to PI3K/mToR inhibitors found to trigger radio-sensitization in the associated PI3K/mToR pathway [67]. The *CTNNB1* gene was prominent in pathways associated with PC, OC, and BC, notifying its importance in HSCs. Further, *BRCA1* was linked to the homologous recombination pathway across cancer types. *MYC*, *STAT3*, and *ESR1* were implicated in the chemical carcinogenesis receptor activation pathway, while the estrogen signaling pathway, a key pathway in HSCs, featured *JUN* and *ESR1*. In OC, the relaxin signaling pathway, which activates pro-oncogenic pathways such as MAPK, WNT, and Notch, also showed *JUN* as a significant contributor [68]. Additionally, *ESR1* and *JUN* were involved in the endocrine resistance pathway in OC and EC, where signaling pathways associated with growth factor receptors, including key hormone-binding receptors, begin to develop resistance to cellular signaling [69]. In BC, *STAT3* and *MYC* play a role in the transcription of microRNAs (miRNAs) implicated in cancer. These dysregulated miRNAs contribute to processes such as proliferative signaling, evasion of growth suppressors, metastasis, and resistance to cell death (Table 4) [70]. A study on gastric cancer cell proliferation inhibition has stated that the Tumor necrosis signaling pathway (TNFα) played a major role in cell proliferation and targeting that could have better effects on cancer prognosis. In this analysis, the JUN gene in OC was present in the TNF signaling pathway indicating its relation with cell proliferation across various cancers [71]. Amidst the well-established regulatory involvement of these genes in the respective cancers, the current study has identified the complex interactive role of these genes upon inducing HSCs, specific to radiation exposure.

Notably, DNA repair genes such as *NBN*,* XRCC3*,* BRCA1*,* BRCA2*,* ATM*,* RAD51* and *CHEK2* were specifically observed in the clusters associated with BC, OC and PC in the current study. Moreover, pathways including homologous recombination and microRNAs in cancer were found to be significantly enriched with DNA repair genes. However, the hub genes exhibited predominant connectivity and central roles within DNA damage and repair associated pathways, emphasizing their potential functional relevance with respect to all the four cancers in this study.

Currently there are no biomarkers identified to predict radio-sensitivity. As radiotherapy is one of the prominent treatment methods utilized, identification of a biomarker is the need of the hour [65]. Identification of radiation associated biomarkers can aid in better understanding of treatment selection for cancer patients based on the radiation sensitivity [67].

The analysis is limited to computational predictions, although the findings need to be validated through experimental research, the findings could still drive the researchers to develop a diagnostic tool which is important in the case of earlier diagnosis of cancer as well as to decide if a patient could be subjected to radiation therapy for treatment. Moreover, since these biomarkers are well established in the associated pathways they can be explored as therapeutic targets in their respective HSCs with experimental validations in the future. The biomarkers can also be studied in other cancers and the experimental evaluation could be conducted contributing to early diagnosis and treatment of cancer, especially HSCs.

## Conclusion

Cancer caused by radiation is becoming an inevitable concern since most of the cancer diagnostic procedures including computed tomography, magnetic resonance imaging, positron emission tomography, and mammography in the medical field are emitting ionizing and photo radiation. The environmental radioactive pollutants, exposure to radiation through occupational means, and exposure to non-ionizing radiation through household appliances are unavoidable, thus it is important to find a biomarker for early diagnosis. The current study has computationally predicted *MYC* and *STAT3* in BC, *CTNNB1* in PC, *JUN*, and *BRCA1* in OC, and *ESR1* in EC as radiation-induced cancer biomarkers. The findings could be lay a base-support for the experimental researchers working on biomarker identification and radiation effects. Additionally, the study contributes to a wider understanding of the pathway mechanisms of cancers induced by radiation.

## Supplementary Information

Below is the link to the electronic supplementary material.

Supplementary Material 1.

## Data Availability

All data generated during the study are included in this article, and were retrieved from the following database. The radiation specifc genes from neoplasms were retrieved from the Comparative Toxicogenomics Database (CTD), 2023 (https://ctdbase.org/), accession ID D009381. The mutated genes list specific breast, ovarian, endometrial and prostate cancer were retrieved from the Cancer Genetics Web database, 2020 (http://www.cancerindex.org/geneweb/). The hypoxic and oxic gene signatures due to radiation exposure were retrieved from the RadiationGeneSigDB at (https://github.com/vmsatya/RadiationGeneSigDB). The hallmark genes up-regulated and down-regulated in response to ultraviolet (UV) radiation were obtained from the Gene Set Enrichment Analysis (GSEA) database (https://www.gsea-msigdb.org/gsea/index.jsp). Constructing a Venn diagram for oxic genes, and hypoxic genes in correlation to the cluster genes was performed using Bioinformatics and evolutionary genomics webserver (BEG), (https://bioinformatics.psb.ugent.be/webtools/Venn/). The functional enrichment analysis and the Gene Ontology (GO) analysis was performed using g: Profiler, 2023 webserver (https://biit.cs.ut.ee/gprofiler/gost). The expression analysis was performed using the TNM plot webserver, 2021 (https://tnmplot.com/analysis/). cBio Cancer Genomics portal (http://cbioportal.org/), v6.3.5 was used to perform survival analysis.

## Glossary

Betweenness: It is a type of centrality parameter in the cytoHubba plugin in Cytoscape that quantifies the importance of nodes based on the shortest paths that pass-through nodes.
Closeness: This centrality parameter measures how close a particular node is to all other nodes in a network.
Degree: The degree of a node could be defined as the number of direct edges it has to other nodes in the network reflecting its dense connectivity in the network.
Edge percolated component (EPC): In biological networks, EPC could be defined as the critical edge, its removal could significantly limit the network’s connectivity. This gives out the nodes that contain critical edges in a network.
Gene ontology: It is the knowledge of the biological domain that provides a set of concepts and relationships to describe the function of the gene concerning biological process (BP), molecular functions (MF), and cellular components (CC).
Hub genes: The genes that are pivotal in a network based on several centralities and topological parameter analyses are referred to as hub genes. These genes are significant to the network’s structure and function.
Hypoxia and Oxic: Hypoxia is the condition characterized by reduced oxygen level in body tissues and the condition in which the body tissues are oxygenated is termed as oxic condition.
Ionizing and non-ionizing radiation: Ionizing radiation consists of high-energy electromagnetic waves that can ionize atoms or molecules by removing electrons from them where whereas electromagnetic radiation that does not carry enough energy to knock out electrons to ionize atoms or molecules is non-ionizing radiation.
Neoplasm: It is an abnormal mass of tissue that arises from uncontrolled cell growth. It can be classified as either benign or malignant tumor.
Radio sensitization: The process by which the cells are made sensitive to radiation is called radio sensitization.
Tumor microenvironment (TME): The environment around a tumor cell that is surrounded by normal cells, molecules, and blood vessels is collectively termed a tumor microenvironment.
Water radiolysis: The process of decomposition of water molecules resulting from ionizing radiation is called water radiolysis.
