# Supplementary material for: Systems biology-driven identification of biomarkers and significant pathways in radiation-induced hormone-sensitive cancers
Source: Discov Oncol. 2025 Nov 4;16:2029. doi: 10.1007/s12672-025-03892-3 (PMC12586841; doi:10.1007/s12672-025-03892-3)
Supplement: Supplementary file 1 — Supplementary Material 1. [file 12672_2025_3892_MOESM1_ESM.pdf]

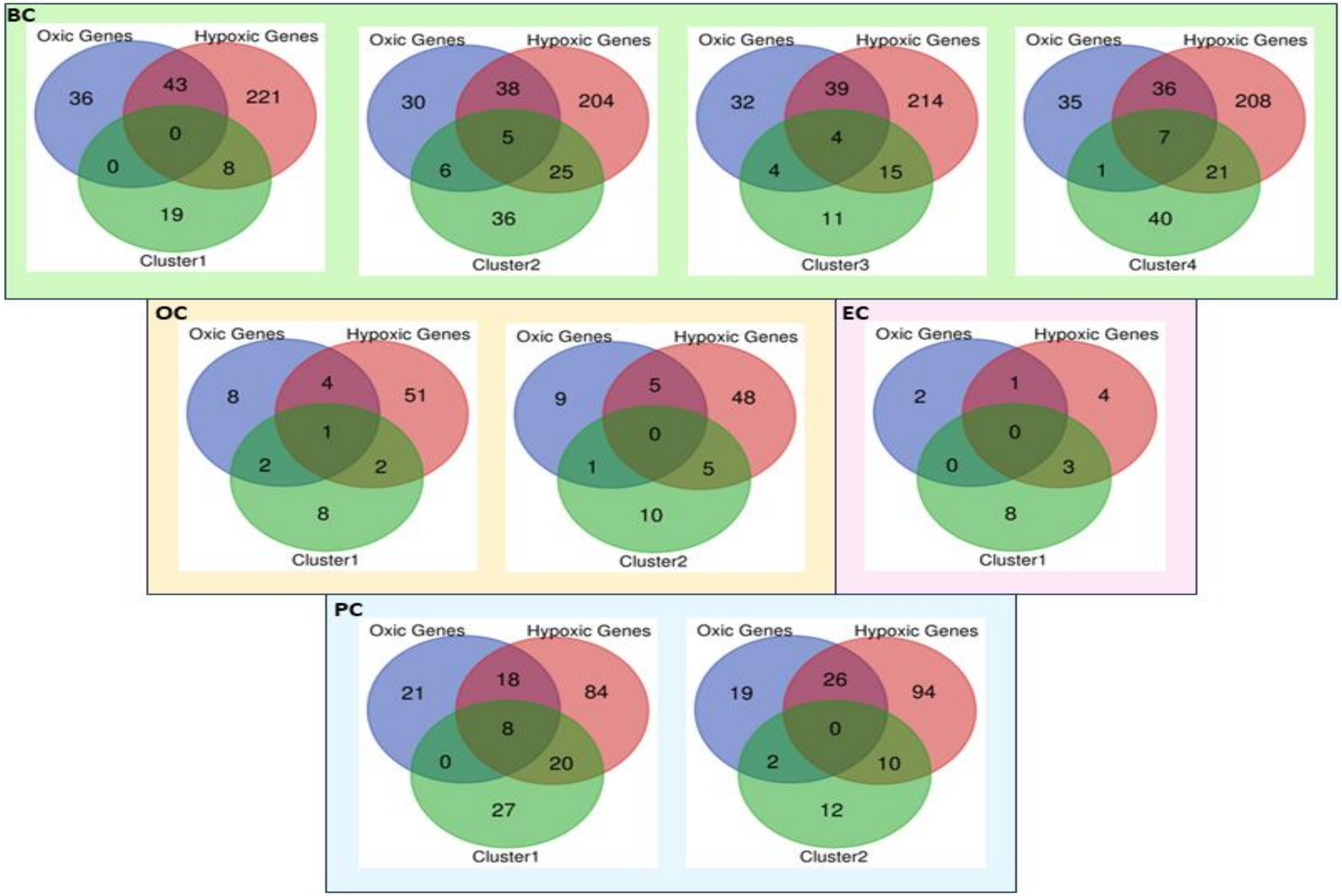

Figure S1: Hypoxic and Oxic gene signatures in HSC clusters. The red circle represents the No. of hypoxic genes, the blue circle represents the No. of oxic genes, and the green circle represents the No of genes in the clusters of each cancer. The Venn diagram on the green background is for clusters of Breast Cancer; the Venn diagram on the yellow background is for clusters of Ovarian Cancer; the Venn diagram on the pink background is for clusters of Endometrial Cancer; the Venn diagram on the blue background is for clusters of Prostate Cancer.

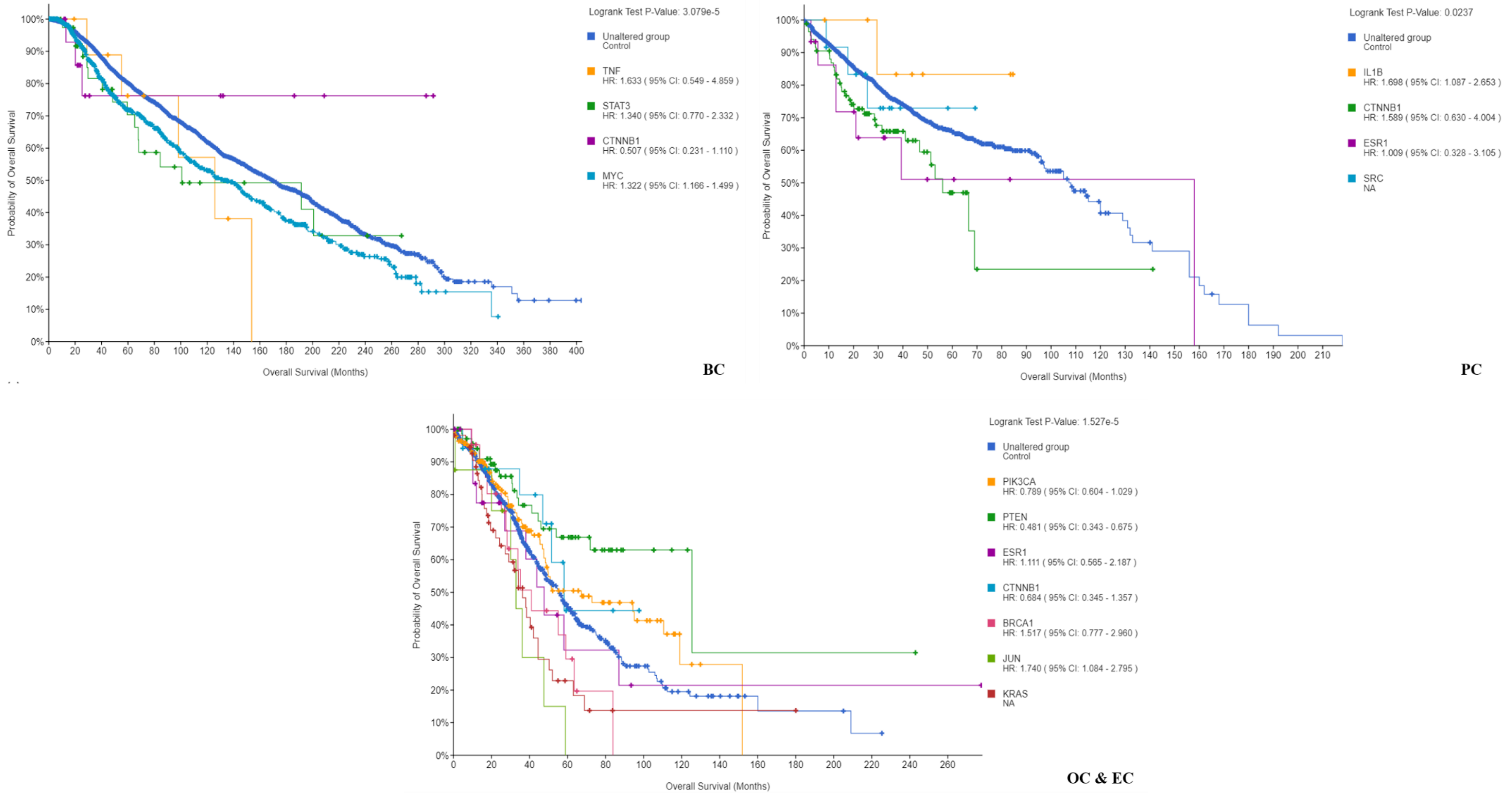

Figure S2: Survival analysis for HSCs. In X axis number of months, a patient could survive in the presence of the gene is shown. The Y axis represents the survival probability for the patients in the presence of the gene, the graph compares the survival between the highly expressing mutated genes against the unaltered genes in BC - Breast Cancer, PC - Prostate Cancer and in OC & EC - Ovarian and Endometrial Cancer.

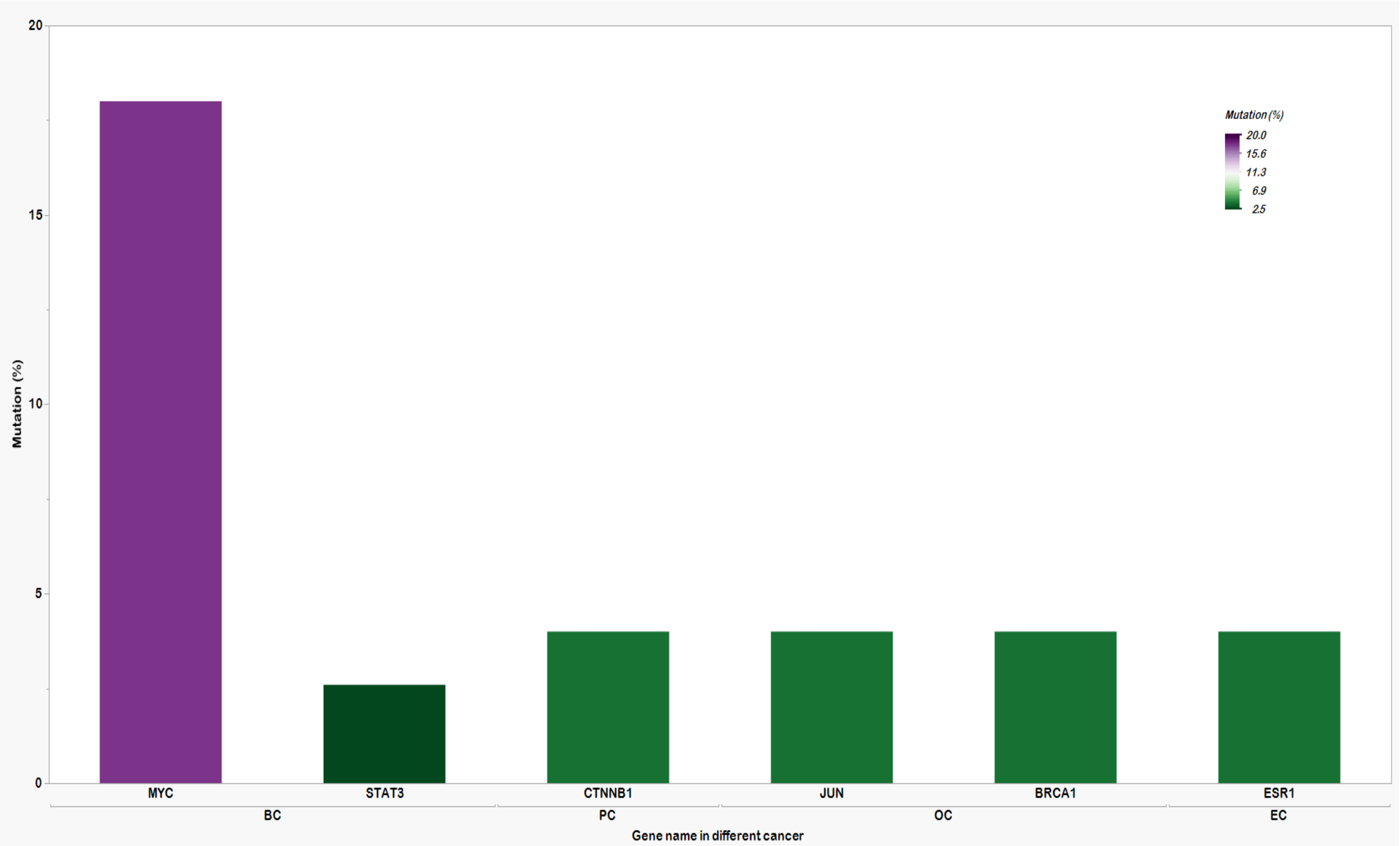

Figure S3: Mutational profile for Biomarker validation. The X-axis represents the biomarkers, and the Y-axis with a color range from green to violet shows the percentage of mutation.

Parameter Summary Table

S. No	Methodology	Parameters used	Result obtained
1	Hub gene identification using cytoHubba	Genes that were in the Top 10 ranks in these parameters (Degree, EPC, Closeness, and betweenness) were selected	BC – 07, PC – 06, OC – 07, EC – 08 and UV – 08 hub genes were obtained
2	Clustering using MCODE	k-means value >3, clustering score ≥ 5.0, and number of nodes > 10 were selected	Obtained 04 clusters
3	Functional Enrichment Analysis and Gene ontologies analysis using g: Profiler	Enrichment ratio based top 10 pathways, statistical significance filtering with p value < 0.05	Top 10 pathways in HSCs and Radiation networks
4	Comparative analysis	Cross verified how many of the hub genes and the cluster genes were involved in oxic and hypoxic conditions in HSCs	BC – 04, PC – 04, OC – 04, and EC – 03 hypoxic hub genes were obtained
5	Expression analysis using TNM plot server	P – value less than 0.5 were considered	The up regulation and down regulation for the concerned genes of the respective cancers were obtained
6	Survival and mutational analysis using c bioportal	Genes with Hazard ratio greater than 1 and mutation percentage less than 5 were considered	The genes important for survival of the cancer were obtained
